# Graphene Facilitated Removal of Labetalol in Laccase-ABTS System: Reaction Efficiency, Pathways and Mechanism

**DOI:** 10.1038/srep21396

**Published:** 2016-02-19

**Authors:** Shipeng Dong, Huifang Xiao, Qingguo Huang, Jian Zhang, Liang Mao, Shixiang Gao

**Affiliations:** 1 State Key Laboratory of Pollution Control and Resource Reuse, School of the Environment, Nanjing University, Nanjing 210093, China; 2 National Laboratory of Solid State Microstructure and Department of Physics, Nanjing University, Nanjing 210093, P. R. China; 3 Department of Crop and Soil Sciences, University of Georgia, Griffin, GA 30223, United States

## Abstract

The widespread occurrence of the beta-blocker labetalol causes environmental health concern. Enzymatic reactions are highly efficient and specific offering biochemical transformation of trace contaminants with short reaction time and little to none energy consumption. Our experiments indicate that labetalol can be effectively transformed by laccase-catalyzed reaction using 2, 2-Azino-bis-(3-ethylbenzothiazoline-6-sulfonic acid) (ABTS) as a mediator, while no significant removal of labetalol can be achieved in the absence of ABTS. A total of three products were identified. It is interesting that the presence of graphene greatly increased the reaction rate while not changed the products. In the presence of 100 μg/L graphene, the pseudo-first-order reaction rate constant was increased ~50 times. We found that the enhancement of graphene is probably attributed to the formation and releasing of ABTS^2+^ which has a much greater reactivity towards labetalol when graphene is present. This study provides fundamental information for laccase-ABTS mediated labetalol reactions and the effect of graphene, which could eventually lead to development of novel methods to control beta-blocker contamination.

As an adrenergic antagonist, beta-blockers are mainly used for the treatment of hypertension, congestive heart failure and abnormal heart rhythms, and more than 100 tons of beta blockers are consumed in Germany alone each year^1^,^2^. Beta blockers were reported to be found ubiquitously in wastewater, surface waters and even in ground water in the ng·L^−1^ to μg·L^−1^ range^3^,^4^,^5^,^6^,^7^,^8^,^9^. It has been reported that beta-blockers are toxic to aquatic organisms by inducing cardiovascular dysfunction^10^,^11^ and affect human cells by reducing viability and inducing apoptosis^12^. Their continuous introduction to the environment is thus potentially harmful to organisms and human. Previous studies have shown that removal of several beta-blockers in traditional sewage treatment plant or wastewater treatment plant is generally below 20%^13^,^14^. As such, beta-blocker may persist in surface waters and groundwater due to their relatively slow rate of elimination from the natural environment^15^,^16^ Certain advanced treatment technologies, such as chlorination^17^, advanced oxidation processes^18^ and photocatalytic degradation^19^,^20^, appear viable for the removal of some beta-blockers. However, these technologies require very high inputs of energy and reagents to bring the trace contaminants from very low levels to even lower safety levels. In addition, hazardous by products can be generated during the oxidation processes. For instance, the chlorination products of Atenolol, a beta-blocker similar to labetalol, can induce phytotoxicity^21^. As such, technologies that can remove beta blocker cost-effectively and safely must therefore be developed. Among all reported beta blockers, labetalol is identified to the most persistent in water treatment, and thus causes a significant concern^21^.

Enzymatic reactions are highly efficient and specific offering biochemical transformation of trace contaminants with short reaction time and little to none energy consumption^22^. Laccase, an oxidase, uses molecular oxygen to initiate catalysis and mediates quick and efficient conversion of natural or anthropogenic chemicals containing phenolic or anilinic moieties^23^. It has been demonstrated that the removal of substrate compounds was enhanced by the addition of a mediator that is comparatively more reactive toward the selected catalyst than the target xenobiotic itself^24^. For example, the presence of natural organic matter engendered the reactions of polychlorinated biphenyls and polyaromatic hydrocarbons that, lacking a phenolic or anilinic subunit, cannot themselves serve as active donors for the enzyme^25^,^26^. In particular, 2,2′-azinobis-(3-ethylbenzothiazoline-6-sulfonate) (ABTS) was reported to be able to expand the substrate specificity of laccase as a redox mediator. Studies have demonstrated that, after inclusion of ABTS, the substrate range of laccase can be extended to nonphenolic subunits of lignin which cannot be transformed by laccase alone^27^. Furthermore, the use of ABTS also significantly increased the removal of endocrine disrupting chemicals bisphenol A (from < 20% increased to ~100%) and nonylphenol (from ~65% increased to ~100%) in the same incubation period^28^.

Graphene has attracted a great attention in recent years for its exceptional electronic quality^29^,^30^. Several groups have demonstrated that graphene sheets show fast electron transfer kinetics and excellent electrocatalytic characteristics^31^,^32^,^33^. Fu and Zhu^32^ reported that graphene oxide effectively mediated the reductive transformation of nitroaromatic compounds and served as the conductor for the electron transfer during the catalytic process. Xue *et al.*^33^ pointed out hemin-graphene conjugates, formed by immobilization of monomeric hemin on graphene, showed excellent catalytic activity, more than 10 times greater than that of the hemin-hydrogel counterpart and 100 times greater than the unsupported hemin. However, the impact of graphene on the enzyme-catalyzed redox reactions has not been studied so far.

We in this study explored the transformation of labetalol during laccase-catalyzed reactions in the presence of ABTS as a mediator, and, in particular, examined the influence of graphene on the reaction efficiency and pathway. It is intriguing that the presence of graphene significantly accelerated the reaction rate, and the mechanism of the reaction rate enhancement was explored.

## Results

### Laccase-mediated labetalol reaction using ABTS as co-substrate

**Figure 1:**
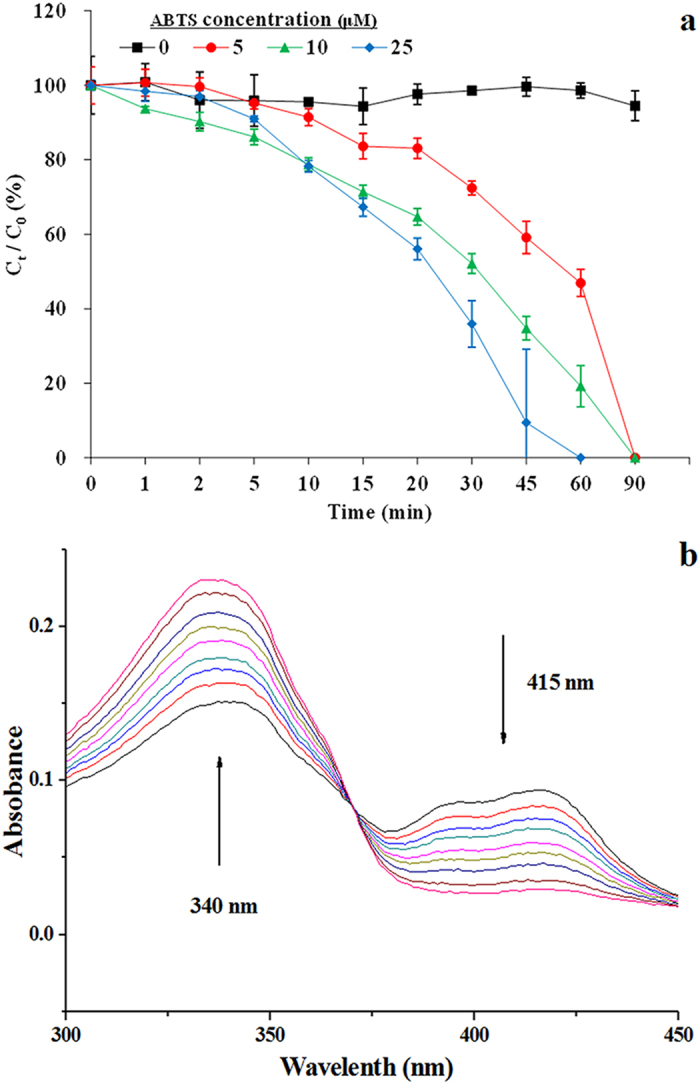
(**a**) Labetalol removal over time after the addition of different concentrations of ABTS in a laccase-mediated reaction system; (**b**) Time based differential UV absorbance of ABTS^•+^ treated by labetalol. Experimental conditions: (**a**), [labetalol]_0_ = 5 μM; [laccase] = 0.1 U mL^−1^, pH 7.0; (**b**), [laccase] = 0.1 U mL^−1^, [ABTS] = 3.1 μM, [ABTS^•+^] = 2.6 μM, [labetalol]_0_ = 5 μM, pH 7.0. Reaction time 30 min. Error bars represent standard deviations (*n* = 3).

Figure 1a presents the labetalol concentrations as a function of time for laccase-mediated reactions utilizing various ABTS concentrations. Results from the control sample (see Fig. 1a) confirm that no significant removal of labetalol was observed in the absence of ABTS. In contrast, appreciable removal of labetalol was achieved with the presence of ABTS. Labetalol removal increases with increasing cosubstrate concentration. The time required to achieve complete labetalol removal was 90 min while the concentration of ABTS was 5 or 10 μM. When 25 μM ABTS was present, the transformation was significantly accelerated, requiring only ∼60 min to achieve complete removal of 5 μM labetalol.

ABTS *per se* is a substrate of laccase, which can be oxidized by laccase to form a stable cation radical (ABTS^•+^)^34^ (see Fig. S1 of Supplementary Information (SI)). However, labetalol is not a direct laccase substrate, therefore we hypothesized that the removal of labetalol in laccase-mediated reactions was likely resulting from the ABTS^•+^ oxidation. As described in Experimental Section, we conducted experiments in which ABTS^•+^ solution was incubated with labetalol and the UV absorbance spectrum was scanned to probe the reaction and the results were shown in Fig. 1b. It is evident in the figure that ABTS^•+^ -specific 414 nm peaks decreased with the incubation time and a chromophore at 340 nm, which is specific to ABTS, was generated^34^. As shown in Fig. S2 of SI, the removal rate of ABTS^•+^ and the yield rate of ABTS is synchronous. After 30 min reaction, 1.79 μM ABTS^•+^ was transformed while 1.62 μM ABTS formed. The concentration of labetalol during the reaction was also tracked and ~1.7 μM labetalol was removed in the reaction. As such, labetalol was oxidized by ABTS^•+^ in the laccase-catalyzed ABTS reaction system and the stoichiometric ratio between labetalol and ABTS^•+^ approaches 1:1. Thus, the reaction can be divided into two steps: step 1, laccase-catalyzed oxidation of ABTS to ABTS^•+^ (see Fig. S1 in SI); step 2, oxidation of labetalol in a subsequent non-enzymatic step by the action of ABTS^•+^ .

### Product identification and reaction pathways

**Figure 2:**
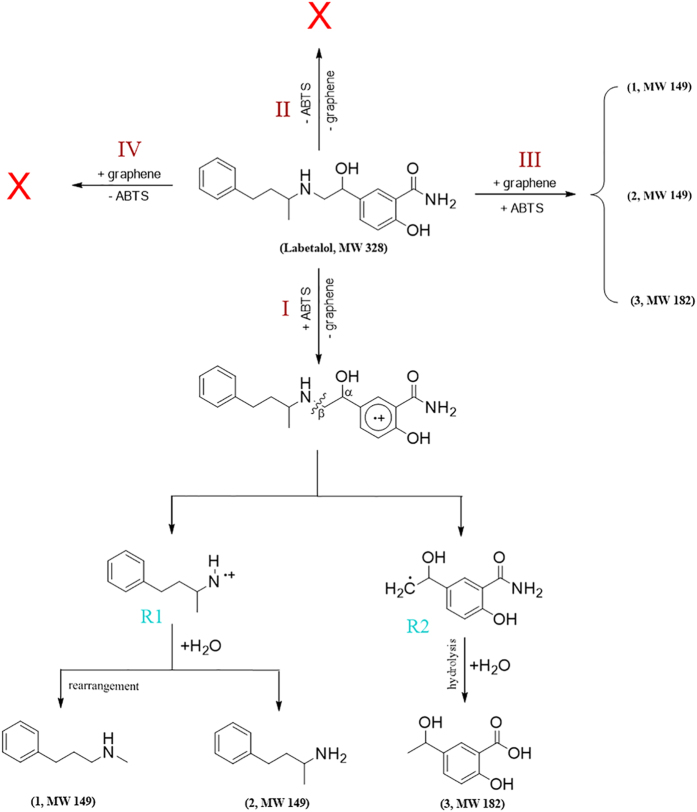
Possible reaction pathways of labetalol in laccase-mediated reaction systems variously containing ABTS and/or graphene, including a reaction system containing ABTS at 5 μM (+ABTS); a system containing neither ABTS (−ABTS) nor graphene (−graphene); a system containing both 5 μM ABTS (+ABTS) and 10 μg L^−1^ graphene (+graphene), and a system containing 10 μg L^−1^ graphene.

Reaction products were identified as means to evaluate possible pathways of laccase-mediated labetalol transformation using ABTS as cosubstrate. Samples for this work were prepared using 5 μM labetalol, 0.1 U mL^−1^ laccase, and 5 μM ABTS; reaction time was 30 min. LC/MS chromatograms for reaction sample using ABTS as cosubstrate contain three peaks that do not appear in the control chromatograms (for samples without laccase or without labetalol) (Fig. S3 in SI). It is assumed that these peaks correspond to products from the laccase-mediated labetalol removal reactions using ABTS as cosubstrate, and their MS spectra were shown in Fig. S4 of SI. Table 1 summarizes all species identified via LC/MS. The proposed reaction pathway is shown in Fig. 2. Initially, labetalol was oxidized by ABTS^•+^ to form a transient intermediate (reaction I). Then the transient intermediate cleaved at the N-C_*β*_ bond and yielded two sterically unstable intermediates (R1 and R2 radical). R1 radical may react with H^+^ to form species 1 (MW = 149); or rearrangement can occur at the nitrogen-connected carbon yielding species 2 (MW = 149). R2 radical become protonated at the carbon with an unpaired electron and the amide group in the radical may undergo hydrolysis to form species 3 (MW = 182).

### Influence of graphene on labetalol reaction in laccase-ABTS system

As shown in Fig. S5 of SI, high quantities of few-layer graphene sheets were successfully synthesized and obtained graphene are mainly consisted of 4-layer graphene. Figure S6 in SI presents the labetalol removal at 60 min in laccase-mediated reactions utilizing various graphene concentrations (ranged from 25 to 250 μg L^−1^). Results from Fig. S6 confirm that no significant removal of labetalol was observed without the ABTS and/or laccase present, although graphene was present in the system. In addition, our preliminary data suggested that the adsorbed labetalol by graphene was fully released after the addition of 1:1 methanol to the solution. As such, we concluded that graphene itself has no impact on the removal of labetalol and cannot serve as the co-substrate like the ABTS does.

**Figure 3:**
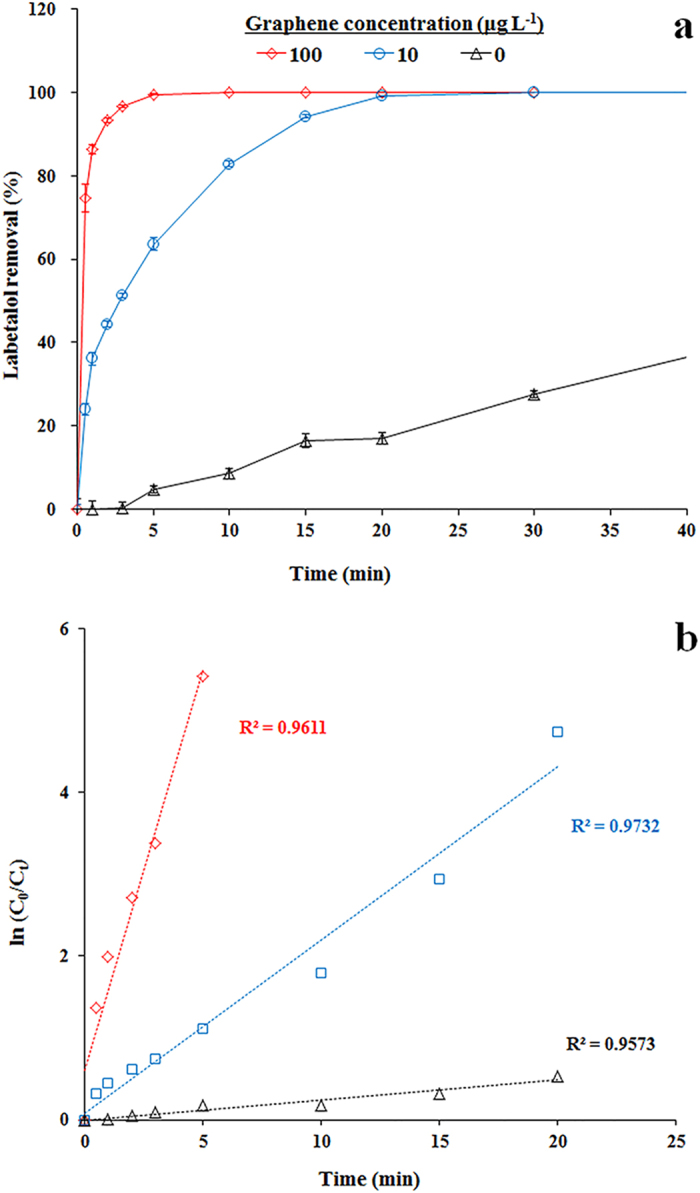
Labetalol removal over time in laccase-ABTS mediated systems with or without the presence of graphene (**a**) and the Pseudo first-order rate plots for labetalol removal (**b**). Experimental conditions were as follows: [labetalol]_0_ = 5 μM, [ABTS] = 5 μM, [laccase] = 0.1 U mL^−1^, pH 7.0. Error bars represent standard deviations (*n* = 3).

Inclusion of graphene in the laccase-ABTS reaction system was further conducted to explore its influence on labetalol removal and the results were presented in Fig. 3a. The experimental results shown in Fig. 3a suggested that the presence of graphene enhanced the removal of labetalol in the laccase-ABTS system. The required time to achieve 100% removal of 5 μM labetalol was 90 min (See Fig. 1a) in the system with 5 μM ABTS only. When 5 μM ABTS and 10 μg L^−1^ graphene were both present in the reaction system, the transformation was significantly accelerated, requiring only ∼20 min to achieve complete removal of 5 μM labetalol. When the concentration of graphene increased to 100 μg L^−1^, even only ~5 min was enough to completely remove 5 μM labetalol. Figure 3b presents the reaction kinetics plotted as ln(C_0_/C_t_) versus time, where C_t_ and C_0_ are the concentrations of labetalol at a given time t and time zero, respectively. The linear regression model fits the data well (R^2^ > 0.96), reflecting that the oxidation of labetalol follows pseudo first-order kinetics. In the control reaction system (in presence of ABTS but absence of graphene), the observed pseudo first-order rate constant (k) is 0.0205 min^−1^; however, the k is significantly enhanced by up to 10.3 and 47.7 times (0.2115 and 0.9771 min^−1^) in the presence of 10 and 100 μg L^−1^ graphene, respectively. In addition, results from products identification confirmed that no additional product except species 1, 2 and 3 yielded in the reaction with both of graphene and ABTS presence (see Fig. 2).

### Proposed enhancement mechanism of graphene

**Figure 4:**
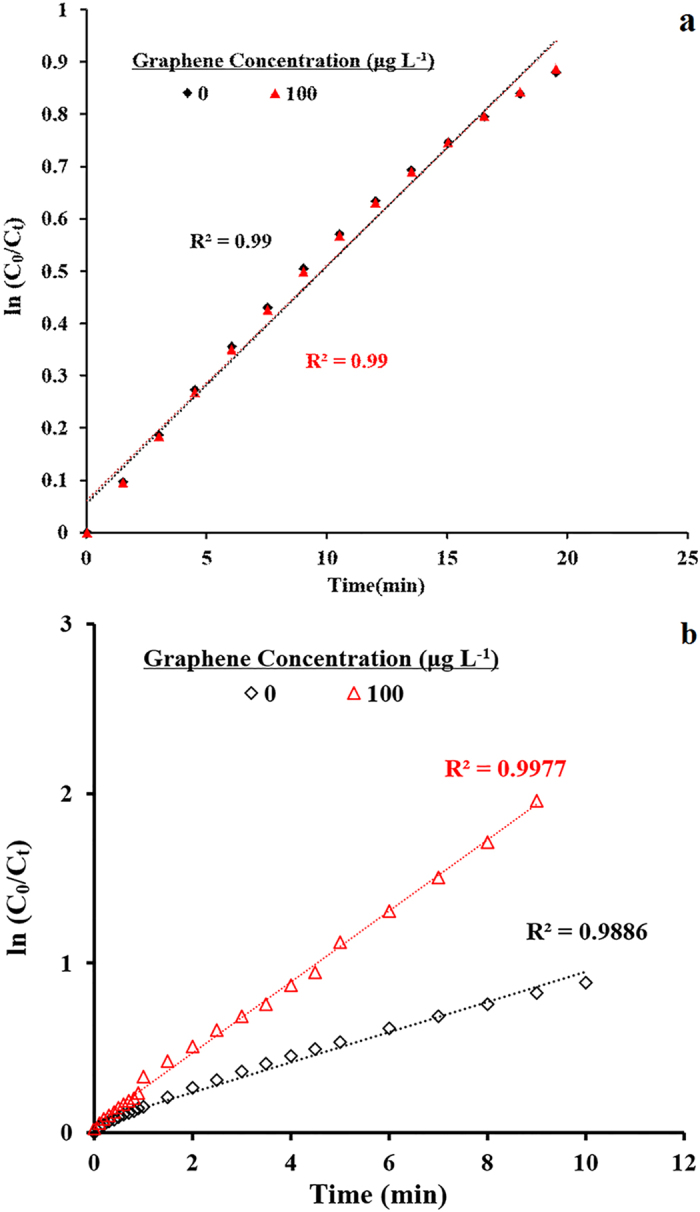
(**a**) Pseudo first-order rate plots of ABTS elimination (equal to the yields of ABTS^•+^), in the laccase-mediated system with or without the presence of graphene. (**b**) Pseudo first-order rate plots of ABTS^•+^ elimination after the addition of labetalol, with or without the presence of graphene. Experimental conditions were as follows: [labetalol]_0_ = 5 μM, [ABTS] = 5 μM, [laccase] = 0.2 U mL^−1^, pH 7.0.

As we mentioned above, the labetalol reaction process in laccase-ABTS system is consisted of two steps that ABTS was oxidized to be ABTS^•+^ (step 1) by which the labetalol was subsequently removed (step 2). The impacts of graphene on the formation rate of ABTS^•+^ (step 1) and the reaction rate between ABTS^•+^ and labetalol (step 2) were explored and the results were presented in Fig. 4. The formation rate of ABTS^•+^ (step 1) had no change (Fig. 4a) while the reaction rate between ABTS^•+^ and labetalol (step 2) was accelerated about 2.36 times (see Fig. 4b) in the presence of 100 μg L^−1^ graphene. As shown in Fig. 3b, rate constant was magnified by 47.7 times when 100 μg L^−1^ graphene presented in the laccase-ABTS system. However, the magnification value for the two steps reaction was only 2.36 times, which is significantly smaller than that of 47.7 times.

As described in Experimental Section, we conducted experiments in which ABTS solution was incubated with laccase and/or graphene and the UV absorbance spectrum was scanned. Differential UV absorbance spectra were calculated using equation 1 to quantify the spectral changes in the system with and without graphene.


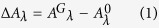


**Figure 5:**
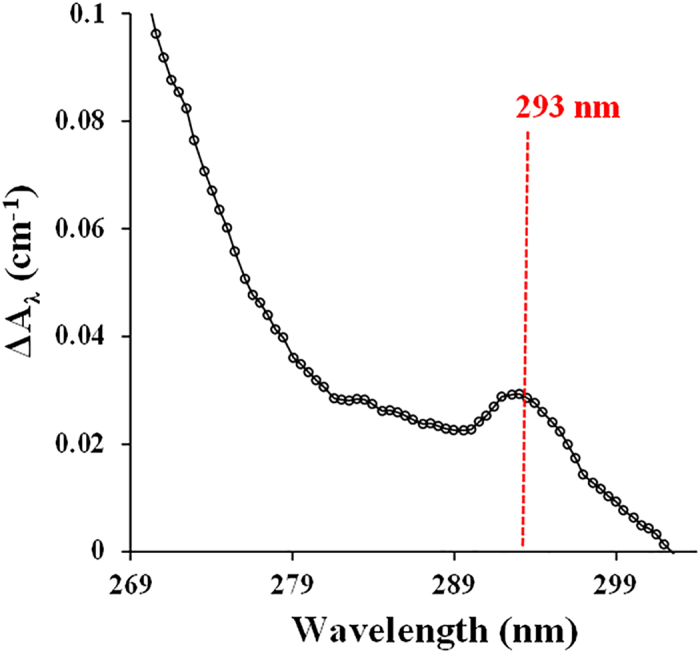
Differential UV absorbance of ABTS treated by laccase with and without the presence of graphene. Experimental conditions: [ABTS] = 10 μM, [graphene] = 100 μg L^−1^, [laccase] = 0.25 U mL^−1^, pH 7.

In equation 1, A^G^_λ_ and A^0^_λ_ are the absorbance of light at wavelength λ for the reaction with and without graphene, respectively, and ΔA_λ_ is the differential UV absorbance at that wavelength. As shown in Fig. 5, a new peak (ΔA_λ_) at 293 nm was found when 100 μg L^−1^ graphene was present in the system. It proved that ABTS could be oxidized to cation radical (ABTS^•+^), and ABTS dication (ABTS^2+^) (as shown in Fig. S7), which has characteristic absorbance at 293 nm. Notably, ABTS^•+^ and graphene has no specific absorbance at this wavelength (see Fig. S9). Thus, the differential UV absorbance (ΔA_λ_) at 293 nm is attributed to the formation of ABTS^2+^. Potassium peroxodisulphate was applied to oxidize ABTS and to further confirm the yields of ABTS^2+^. After 4 min reaction, ABTS (λ = 340 nm) was rapidly transformed to ABTS^•+^ (λ = 414 nm) in the solution (see Fig. S8). As the reaction progressed, the peak at 414 nm gradually decreased with arising of an obvious peak at 293 nm, suggesting the conversion of ABTS^•+^ to ABTS^2+^ 35.

**Figure 6:**
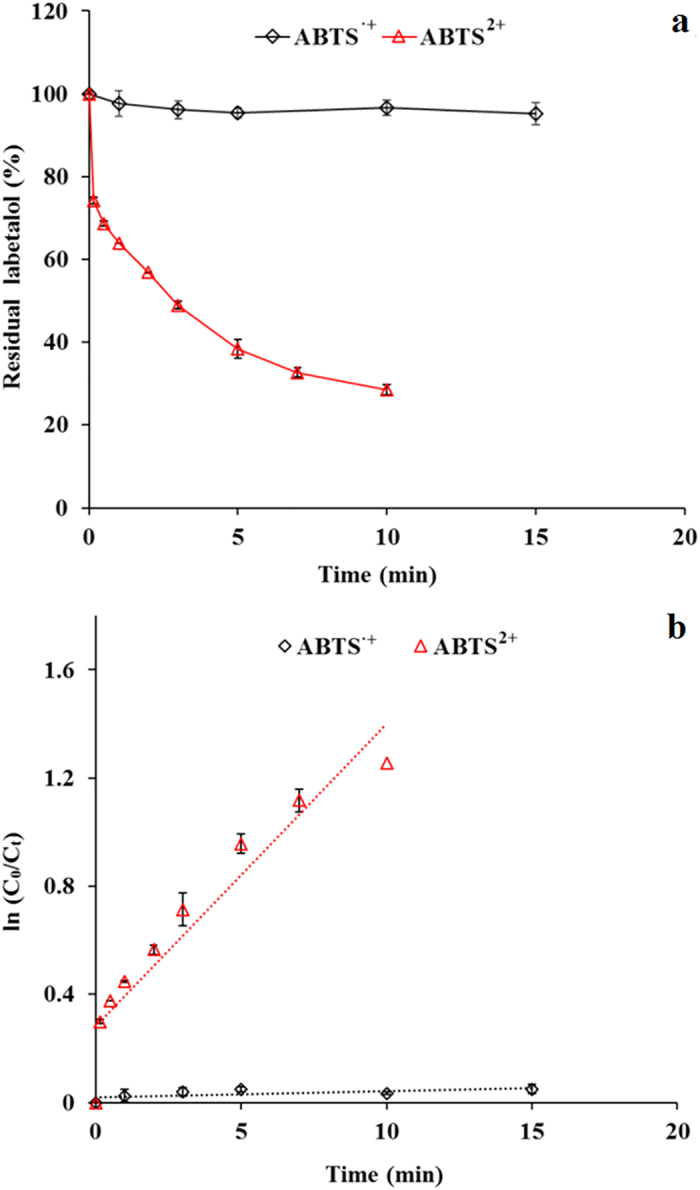
(**a**) Labetalol removal over time using ABTS^•+^ or ABTS^2+^ as oxidants. (**b**) Pseudo first-order rate plots for labetalol removal using ABTS^•+^ or ABTS^2+^ as oxidants. Experimental conditions were as follows: [labetalol]_0_ = 5 μM, [ABTS^•+^] = 5 μM, [ABTS^2+^] = 5 μM, pH 7.0.

Labetalol was cultured with ABTS^2+^ solution and the experimental results in Fig. 6a suggested that labetalol was rapidly removed by ABTS^2+^ and the required time to achieve ~75% removal of 5 μM labetalol was only 10 min. In addition, the removal of labetalol by ABTS^2+^ roughly followed first-order decay (C = C_0_e^−kt^). When ABTS^•+^ was used as oxidant, 5 μM labetalol was only slightly removed (~10%) after 15 min reaction (see Fig. 6a). The pseudo first-order kinetics for labetalol reaction with ABTS^2+^/ABTS^•+^ was obtained and presented in Fig. 6b. In the ABTS^•+^ reaction system, the observed k is 0.0022 min^−1^; however, the k is significantly enhanced by up to 50.8 times (0.1119) in the ABTS^2+^ reaction system. As such, graphene’s enhancement to labetalol removal in the laccase-ABTS reaction is attributed to that the presence of graphene resulted in the yield of ABTS^2+^ , which has much greater reactivity towards labetalol.

## Discussion

The widespread occurrence of the beta-blocker labetalol causes environmental health concern. Our experiments suggest that labetalol was effectively transformed by laccase-catalyzed reaction using ABTS as a mediator, which provided fundamental information for laccase-ABTS catalyzed labetalol reactions and eventually lead to development of novel methods to control beta-blocker contamination. The environmental implications of graphene have received much attention, however, little is known about how graphene affect the enzymatic reactions, which have been examined as a potential means for the removal of trace organic contaminants in water/wastewater.

**Figure 7:**
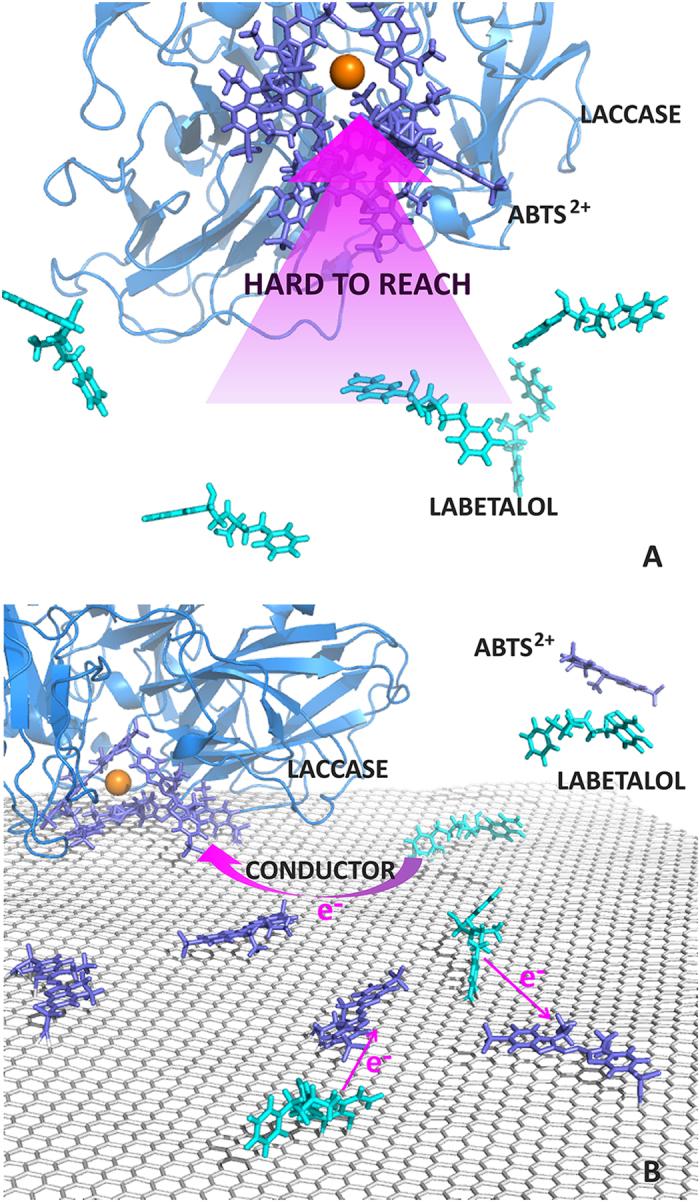
Proposed mechanism of graphene’s enhancement in laccase-ABTS reaction system. (**A**) The positive ABTS^2+^ was trapped in active site of laccase (negative) and hard to reach labetalol; (**B**) Parts of the trapped ABTS^2+^ were released by graphene absorption and the released ABTS^2+^ can thus react with labetalol. And also, the graphene may act as a conductor transferring the electron from labetalol to the unreleased ABTS^2+^ in the active site of laccase to make the reaction occur.

The presence of graphene was found to greatly increase the reaction rate while not change the products. The enhancement was proposed that the presence of graphene released the ABTS^2+^ which has a much greater reactivity towards labetalol. The schematic representation of the proposed mechanism of graphene’s enhancement in laccase-ABTS reaction system was presented in Fig. 7. At pH 7 (reaction pH in this study), the active site of laccase was negatively charged as the acidic isoelectric point of laccase was around pH 4.0^36^. ABTS^2+^ with positive charges (see Fig. S7) was thus bind or trapped in the active site and had no chance to touch and react with labetalol (see Fig. 7A). With the presence of graphene, parts of the trapped ABTS^2+^ were released by graphene absorption and become detectable in the solution by UV (see Fig. 5). The released ABTS^2+^ can thus react with labetalol. And also, the graphene may act as a conductor transferring the electron from labetalol to the unreleased ABTS^2+^ in the active site of laccase to make the reaction occur (see Fig. 7B). However, due to the trapping of ABTS^2+^ by laccase, ABTS^2+^ may not be reduced to its ground state, which may explain the consumption of ABTS in the laccase-mediated reaction. More research should be conducted to explore the detailed mechanism.

In conclusion, this is to our best knowledge the first report of few-layer graphene influencing on the enzyme-catalyzed redox reactions by drastically increasing catalytic activity. Here we proposed a possible mechanism on the basis of the above results. The formation and releasing of ABTS^2+^ which has a much greater reactivity towards labetalol may contribute to the performance enhancement with presence of graphene. Nonetheless, further work is still required to investigate the hypothesis and the mechanism of graphene induced formation of ABTS^2+^ . The results of this study and related studies^33^,^34^,^35^, furthermore, indicate that graphene is possibly a promising matrix to be used in enzyme immobilization for the strong catalytic effects and large surface area of graphene. However, whether graphene has the ability to improve the performance of the immobilized enzyme is still unclear, which is an intriguing topic to be investigated. It may not be certain yet regarding the safety of using graphene in pollutants control. Feng *et al.*^37^ investigated the fate of graphene in water solution and found that graphene was effectively degraded and completely transformed into CO_2_ by Fenton reaction. However, more research should still be conducted (i.e. how to remove graphene from water) for possible future application of graphene in water treatment.

Overall, the outstanding catalytic reinforcement effect makes graphene an excellent candidate for signal transmission and reactivity magnification in enzyme engineering and biological devices development, and can greatly expand the range of applications of graphene-based nanocomposites.

## Methods

### Materials

All reagents were of ACS grade or higher. Labetalol hydrochloride, ABTS and laccase from *Trametes versicolor* was purchased from Sigma-Aldrich (St. Louis, MO). The molecular structure of ABTS was presented in SI. Four-layer graphene was synthesized using the same method employed in our earlier study^38^ and the detail was presented in SI.

### Enzyme activity assay

Laccase was freshly prepared and assayed for activity before each experiment. A colorimetric assay was used to quantify the activity of the laccase enzyme, in which ABTS was oxidized by laccase catalysis to ABTS^•+^ which strong absorbance at 414 nm was measured for quantification (Cary®50, Varian, Inc.)^39^. The enzyme activity assay medium was made up of 1.9 mL sodium acetate buffer (10 mM, pH 4.6), 100 μL fresh laccase solution, and 1 mL ABTS (1 mM). One enzyme unit corresponds to the amount of laccase that oxidizes 1 μmol ABTS per min.

The assay protocol described above was also modified and used to explore the influence of labetalol on the conversion of ABTS^•+^ (see Fig. S1 of SI). The experiments were performed in 25 mL conical flasks as batch reactors under room temperature. Each reactor contained 10 mL solution containing 5 μM ABTS and 0.1 U mL^−1^ laccase buffered at pH 7.0. At the end of 30 min incubation, 5 mL methanol was added to the solution to inactivate laccase^40^, after which a 3 mL sample was transferred to a 1-cm quartz cuvette and then 30 μL labetalol (5 μM, final concentration) was added to the reaction solution and mixed. At each of the following time intervals: 0, 2, 4, 6, 10, 14, 20, 24, 30 min, the UV absorbance spectrum of the solution in the cuvette was scanned using a Cary®50 spectrophotometer. A 0.5 mL aliquot of the reaction solution was sampled at each of the reaction times 0 and 30 min. Labetalol concentration remaining in the solution was analyzed using HPLC. Details of the HPLC method for labetalol analysis are shown in SI.

### Assessment of labetalol removal at varying reaction conditions

Experiments were conducted in glass test-tube batch reactors, which were incubated on a rotary shaker at 150 rpm. A series of reaction media (3 mL) were prepared in a phosphate buffer solution (PBS, 10 mM, pH 7.0) containing 5 μM labetalol, 0.1 U mL^−1^ laccase and either ABTS (ranged from 0 to 25 μM) or graphene (ranged from 25 to 250 mg L^−1^). Laccase was added to each reactor as the last component to initiate the reaction, following which the reactors were dosed with 3 mL methanol at pre-specified intervals (1, 2, 5, 10, 15, 20, 30, 45, 60 and 90 min) (to terminate the reaction) and sacrificed to measure labetalol concentration. The mixture of reaction medium and methanol was sampled for HPLC analysis. Three replicate experiments were performed for each reaction condition. Identical reactors without laccase or co-substrate were used as controls.

Experiments were also carried out using the same reactor setup and procedure described above to examine how the presence of graphene may influence laccase-ABTS catalyzed labetalol removal. A graphene stock solution was prepared and 0.4 mL of the stock solution was added into 19.6 mL reaction solution containing 0.1 U mL^−1^ laccase, 5 μM labetalol and 5 μM ABTS, such that the total volume in each reactor was 20 mL and the final graphene concentration was either 10 or 100 μg L^−1^. Nine sets of reactors were prepared in triplicate. At pre-specified intervals (1, 2, 5, 10, 15, 20, 30, 45 and 60 min), 1 mL reaction solution was sampled to centrifuge tube that contained 1 mL methanol (to terminate reaction) and then subjected to 10 min of centrifugation at 47000 g. After centrifugation, the solution was sampled to measure labetalol concentration using HPLC. Reactors that had laccase or ABTS absent served as controls.

In order to make graphene stock solution (5 mg L^−1^), a 500 mL beaker containing 1 mg graphene and 200 mL of water was placed in ice-water bath. The solution was sonicated for 10 h with the probe tip of ultrasonic processor (100 W, P = 7.52 J/s)^41^ approximately 0.4 cm from the bottom of the beaker. Our previous results^38^ suggested that > 95% of the graphene was suspended in this stock solution at 24 h.

### Product identification

Samples for product identification were prepared in a 250 mL flask reactor containing 100 mL of reaction solution comprising 5 μM labetalol, ABTS (5 μM), 0.1 U mL^−1^ laccase, and with or without the presence of 10 μg L^−1^ graphene. These reactors were incubated on a rotary shaker for 30 min. At sampling time, product mixtures were concentrated using solid phase extraction (200 mg/6 mL Oasis HLB prepackaged cartridges purchased from Waters) prior to LC/MS characterization. Blank samples that did not contain laccase or labetalol were also analyzed. Detailed LC/MS characterization was presented in SI.

### UV-spectrum of ABTS in laccase-mediated system with and without graphene presence

The experiment was performed in 3 mL test-tube as reactor under room temperature. The tube contained 3 mL solution containing 10 μM ABTS, 0.25 U mL^−1^ laccase, 100 μg L^−1^ graphene and buffered at pH 7. At the end of 20-min incubation, 0.6 mL acetonitrile was added to the solution, after which a 2 mL sample was transferred to a 1-cm quartz cuvette. The UV absorbance spectrum of the solution in the cuvette was scanned using a Cary®50 spectrophotometer. Baseline correction was applied to exclude graphene absorbance as a background. Identical reactor without graphene was used as control.

### Labetalol reaction with ABTS^•+^/ABTS^2+^

Reactions of labetalol mediated by ABTS^•+^/ABTS^2+^ were conducted in 50 mL flasks as batch reactors. Each reactor contained 15 mL of PBS solution, containing 5 μM ABTS^•+^/ABTS^2+^ and 5 μM labetalol, which was incubated on a rotary shaker at 150 rpm. The method to prepare ABTS^•+^/ABTS^2+^ was described below. Reactors with ABTS^•+^/ABTS^2+^ absent were also prepared to serve as controls. Experiments were performed for each reaction condition in triplicate. A 0.5 mL aliquot of the reaction solution was sampled at each of the reaction times 30 s, 1, 2, 3, 5, 7, 10 and 15 min and mixed with 50 μL NaOH (1 M) to terminate the reaction. Then labetalol concentration was measured using HPLC.

ABTS^•+^ was prepared in 5 mL of PBS solution containing 0.1 U mL^−1^ laccase and 10 μM ABTS. After 60 min, the reaction was quenched by the addition of HCl (2 M) to adjust the pH to be lower than 1. ABTS^2+^ was prepared by the reaction between ABTS and excessive potassium peroxodisulphate which can ensure the stability of the oxidized state of the dication^39^. The reaction was conducted in 25 mL flask that contained 5 mL of peroxodisulphate solution (200 mM) and was incubated on a rotary shaker at 300 rpm. A 50 μL of ABTS stock solution (50 mM) was added into the reactor for four times in sequence (200 μL in total). After the last addition of ABTS, a red-brown brittle precipitate was formed and separated by centrifuge (10 min, 1400 g). The separated precipitate was washed using 5 mL DI water for three times. The precipitate of ABTS^2+^ was resuspended in 5 mL water and kept for further experiments.

## Additional Information

**How to cite this article**: Dong, S. *et al.* Graphene Facilitated Removal of Labetalol in Laccase-ABTS System: Reaction Efficiency, Pathways and Mechanism. *Sci. Rep.* **6**, 21396; doi: 10.1038/srep21396 (2016).

## Supplementary Material

Supplementary Information

## Figures and Tables

**Table 1:** HPLC Separation and MS Characterization of SPE Extracts of Samples Taken after 30 min of Reaction^a^.

retention time (LC min)	molecular ion^b^	molecular weight	possible structure^c^
6.6	150	149	1
9.6	150	149	2
12.5	329	328	labetalol
13.0	183	182	3

^a^The reaction condition, SPE procedure, and LC/MS condition are described in the Experimental Section of the SI.

^b^Detailed masses and selected structures for the molecular ions are shown in Fig. S5 of the SI.

^c^Detailed structures are shown in Fig. S5.
