# Supplementary material for: Graphene Facilitated Removal of Labetalol in Laccase-ABTS System: Reaction Efficiency, Pathways and Mechanism
Source: Sci Rep. 2016 Feb 19;6:21396. doi: 10.1038/srep21396 (PMC4759553; doi:10.1038/srep21396)
Supplement: Supplementary Information [file srep21396-s1.doc]

**Supplementary Information**

**Graphene Facilitated Removal of Labetalol in Laccase-Mediated System: Reaction Efficiency, Pathways and Mechanism**

# Shipeng Dong^†^, Huifang Xiao^‡^, Qingguo Huang ^§^, Jian Zhang ^‡^, Shixiang Gao ^†^, Liang Mao ^†,*^

^†^ State Key Laboratory of Pollution Control and Resource Reuse, School of the Environment; ^‡^ National Laboratory of Solid State Microstructure and Department of Physics, Nanjing University, Nanjing 210093, P. R. China

^§^ Department of Crop and Soil Sciences, University of Georgia, Griffin, GA 30223, United States

^*^Address correspondence to L. Mao, State Key Laboratory of Pollution Control and Resource Reuse, School of the Environment, Nanjing University, Nanjing 210093, P. R. China. Telephone: (86)25-89680393. Fax: (86)25-89680393. E-mail: lmao@nju.edu.cn.

**I. METHOD**

**Graphene synthesis and characterization.** Twenty mL of an ethanol solution containing dodecylamine (5.000 g) was added into 80 mL of a mixed aqueous solution of FeCl_2_·4H_2_O (1.590 g) and NH_4_H_2_PO_4_ (0.900 g) at 50°C. The solid product was collected by centrifugation and dried under vacuum at 40°C for 12 h. The solid was then heated at 700°C for another 12 h under argon. After cooling, the black powder and 37% hydrochloric acid were transferred into a Teflon-lined autoclave and sealed before being heated in an oven at 180°C for 24 h. The black solid product was collected by centrifugation, and then washed by water and anhydrous ethanol over ten additional times to ensure the purity of the graphene. The following equipment were conducted to characterize the synthesized graphene.

Transmission electron microscopy (TEM) and High resolution TEM (HRTEM) measurements were conducted with a JEM-2010 electron microscope, using an accelerating voltage of 200 kV. Raman spectroscopic analysis was performed with a Renishaw InVia system utilizing a 514 nm incident radiation. Scanning electron microscopy (SEM) measurements were conducted with a Hitachi S4800 instrument, using an accelerating voltage of 10 kV. TEM, HRTEM, Raman spectroscopy, and SEM analyses were performed for both the graphene powder and the sonicated graphene. X-ray photoelectron spectroscopy (XPS) measurements of graphene powder were performed on a PHI 5000 VersaProbe with a monochromatic Al Ka X-ray source. Nitrogen sorption isotherms of the graphene powder were collected at 77 K using Micromeritics ASAP2020 equipment. Brunauer-Emmett-Teller (BET) and BJH models are respectively used for specific surface area and porosity evaluation. This method has been published in our earlier paper[^1^](#_ENREF_1).

**HPLC analysis.** An Agilent 1200 High Performance Liquid Chromatography (HPLC) equipped with a fluorescence detector was used to quantify the labetalol. The separation was performed on an Agilent XDB-C18 column (5 μm, 250 mm × 4.6 mm). Injection volume was 20 µL, and the excitation and emission wavelengths were 210 and 420 nm, respectively, for the fluorescent detector. The mobile phase was made up of 75% acetonitrile and 25% water (with 0.5% triethylamine), and eluted at 1 mL min^-1^.

**LC/MS analysis.** LC/MS analysis was carried out on a Thermo liquid chromatograph connected to a Thermo LCQ Advantages (Quest LCQ Duo, USA) mass spectrometer through an ESI interface. The LC separation was achieved using an Agilent XDB-C18 column (5 μm, 150 mm×4.6 mm). The mobile phase was made up of acetonitrile and water, and eluted at 0.2 mL min-1 using a linear gradient program: 0 min, 5% acetonitrile and 30min, 75% acetonitrile. The mass spectrometer was operated in positive ionization mode over the range m/z = 50-1000. The mass spectrometer was set at 4.5 kV capillary voltage, 25 V cone voltage, 300 °C desolvation temperature and 120 °C source temperature. Molecular weight (MW) was assigned on the basis of the pseudomolecular ions [M+H]^+^. The system operated in selected reaction monitoring mode. The molecular ions obtained from full scan mode were chosen as the precursor ions.

**II. Catalytic cycle of laccase-catalyzed ABTS reaction system**


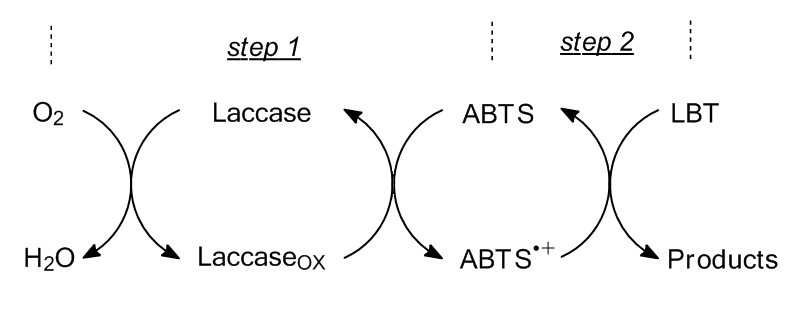


**Figure S1.** Schematic depiction of laccase catalytic cycle and the successive oxidation of the ABTS and labetalol (LBT) [^2^](#_ENREF_2).

**III. ABTS and ABTS^•+^ concentrations over time after the addition of labetalol**


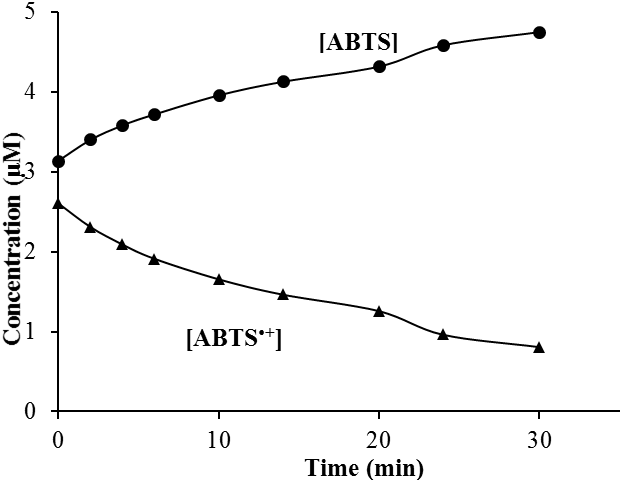


**Figure S2.** ABTS and ABTS^•+^ concentrations over time after the addition of labetalol. Reaction system initially contained 0.1 U mL^-1^ laccase and 10 μM ABTS and was incubated for 30 min to make the ABTS partly transformed to be ABTS^•+^; then 5 mL methanol was added to the solution to make the laccase inactivated and then 5 μM labetalol was finally added.

**IV. LC/MS spectra of the products resulting from the laccase-mediated labetalol reaction using ABTS as the co-substrate**

**Figure S3.** LC spectra of the products resulting from the laccase-mediated labetalol reaction using ABTS as the co-substrate . Initial labetalol concentration is 5 μM; Laccase 0.1 U mL^-1^, pH 7.0, reaction time 30 min, ESI+.

**V. Mass spectra resulting from LC/MS analysis and their possible structures**

**Figure S4.** Mass spectra resulting from LC/MS analysis and their possible structures.

**VI. Graphene characterization**

A TEM image of the as-prepared graphene shown in Figure S6a displays the typical crumpled nanosheets of graphene. HRTEM image shown in Figure S6b indicates that the graphene mainly consisted of 4 layers and the interlayer distance is about 0.344 nm, which is the interlayer distance of graphite. TEM images confirmed that sonication did not change the morphology of the graphene. The marked strong D, G and 2D bands in the Raman spectra (Figure S6c) is well indexed to graphene with multi-layers structure. Raman spectroscopic analysis revealed that there was no change in the Raman spectra bands for the graphene after sonication. A specific surface area of 660 m^2^/g was obtained from the nitrogen sorption/desorption isotherms of the multilayer graphene (Figure S6d) according to the BET model. Considering the specific surface area of the single-layer graphene (2630 m^2^/g)[^7^](#_ENREF_7), the obtained graphene are mainly consisted of 4-layer graphene, in accord with the HRTEM results.

**e**

**b**

**c**

**d**

**a**

**f**

**Figure S5.** Graphene characterization: (a) TEM image, (b) HRTEM image, (c) SEM image, (d) Raman spectra, (e) Nitrogen adsorption/desorption isotherms (inset shows pore-size distribution plot calculated by the BJH formula in the desorption branch isotherm), and (f) XPS spectrum of the graphene powder.

**VII. Effect of graphene on laccase-catalyzed labetalol reaction**


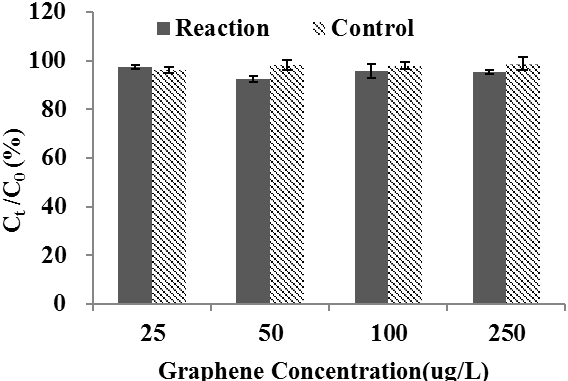


**Figure S6.** Laccase-catalyzed labetalol removal in systems containing different concentrations of graphene, without the presence of ABTS. Samples that had laccase absent served as control. Experimental conditions were as follows: [labetalol]_0_ = 5 μM; [laccase] = 0.1 U mL^-1^; reaction time, 60 min; pH 7.0. Error bars represent standard deviations (*n* = 3).

**VIII. Redox transformations of ABTS.**

**Figure S7.** The redox transformations of ABTS to cation radical (ABTS^•+^) and dication (ABTS^2+^) forms [^8^](#_ENREF_8).


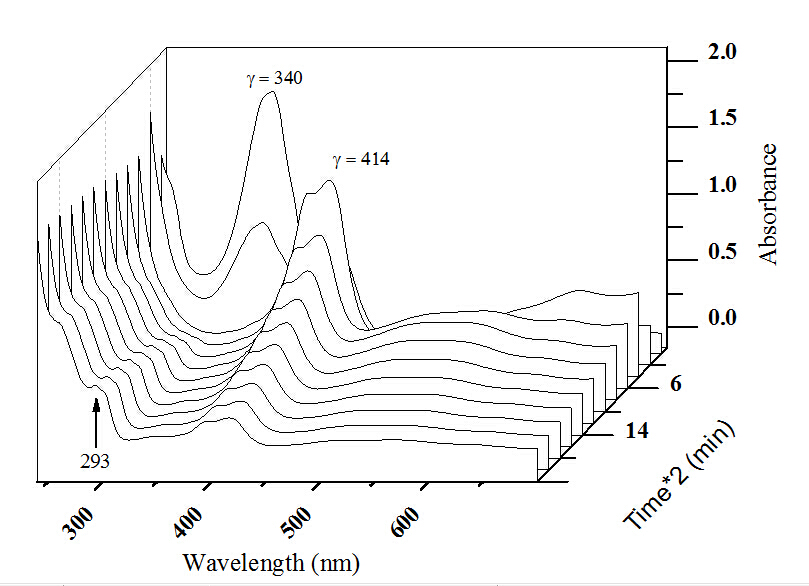


λ=340

λ=414

**Figure S8**. UV-vis spectrum of the ABTS transformation over time. The spectra were acquired each 2 min during 20 min of reaction. Experimental conditions were as follows: [ABTS] = 50 μM, [K_2_S_2_O_8_] = 20 mM, pH 4.6.


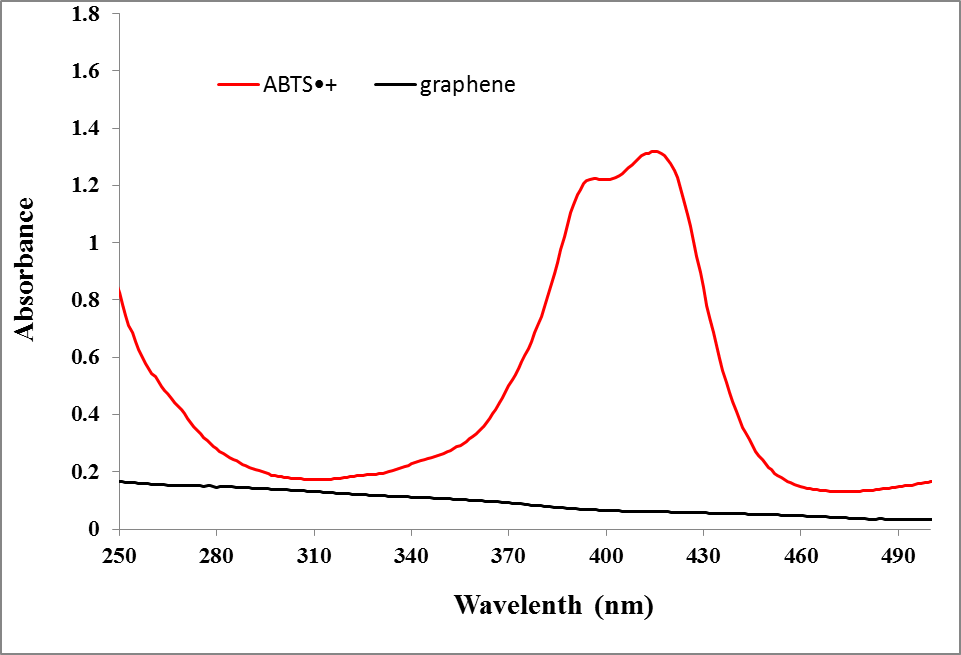


**Figure S9**. UV-vis spectrum of ABTS^•+^ and graphene.

**Reference**

1 Guo, X. *et al.* Sandwich-like LiFePO4/graphene hybrid nanosheets: In situ catalytic graphitization and their high-rate performance for lithium ion batteries. *J. Mater. Chem. A.* **1**, 11534-11538 (2013).

2 Rochefort, D., Leech, D. & Bourbonnais, R. Electron transfer mediator systems for bleaching of paper pulp. *Green Chem.* **6**, 14-24 (2004).

3 Deng, D. *et al.* Toward N-Doped Graphene via Solvothermal Synthesis. *Chem. Mater.* **23**, 1188-1193 (2011).

4 Dresselhaus, M. S. & Dresselhaus, G. Intercalation compounds of graphite. *Adv. Phys.* **30**, 139-326 (1981).

5 Allen, M. J., Tung, V. C. & Kaner, R. B. Honeycomb Carbon: A Review of Graphene. *Chem. Rev.* **110**, 132-145 (2010).

6 Gupta, A., Chen, G., Joshi, P., Tadigadapa, S. & Eklund. Raman Scattering from High-Frequency Phonons in Supported n-Graphene Layer Films. *Nano letters.* **6**, 2667-2673 (2006).

7 Stoller, M. D., Park, S., Zhu, Y., An, J. & Ruoff, R. S. Graphene-Based Ultracapacitors. *Nano letters.* **8**, 3498-3502 (2008).

8 Solis-Oba, M., Ugalde-Saldivar, V. M., Gonzalez, I. & Viniegra-Gonzalez, G. An electrochemical-spectrophotometrical study of the oxidized forms of the mediator 2,2 '-azino-bis-(3-ethylbenzothiazoline-6-sulfonic acid) produced by immobilized laccase. *J. Electroanal. Chem.* **579**, 59-66 (2005).
